# Assessing the Validity of Self-Rated Health with the Short Physical Performance Battery: A Cross-Sectional Analysis of the International Mobility in Aging Study

**DOI:** 10.1371/journal.pone.0153855

**Published:** 2016-04-18

**Authors:** Mario U. Pérez-Zepeda, Emmanuelle Belanger, Maria–Victoria Zunzunegui, Susan Phillips, Alban Ylli, Jack Guralnik

**Affiliations:** 1 Geriatric and Epidemiological Research Department, Research Division, Instituto Nacional de Geriatría, Mexico City, Mexico; 2 Public Health Research Institute (IRSPUM), Department of Social and Preventive Medicine, Université de Montréal, Montréal, Québec, Canada; 3 Centre for Studies in Primary Care, Queen's University, Kingston, Ontario, Canada; 4 Medicine Faculty, University of Tirana, Tirana, Albania; 5 Department of Epidemiology and Public Health, University of Maryland School of Medicine, Baltimore, Maryland, United States of America; Children's National Medical Center, Washington, UNITED STATES

## Abstract

**Objective:**

The aim of this study was to explore the validity of self-rated health across different populations of older adults, when compared to the Short Physical Performance Battery.

**Design:**

Cross-sectional analysis of the International Mobility in Aging Study.

**Setting:**

Five locations: Saint-Hyacinthe and Kingston (Canada), Tirana (Albania), Manizales (Colombia), and Natal (Brazil).

**Participants:**

Older adults between 65 and 74 years old (n = 1,995).

**Methods:**

The Short Physical Performance Battery (SPPB) was used to measure physical performance. Self-rated health was assessed with one single five-point question. Linear trends between SPPB scores and self-rated health were tested separately for men and women at each of the five international study sites. Poor physical performance (independent variable) (SPPB less than 8) was used in logistic regression models of self-rated health (dependent variable), adjusting for potential covariates. All analyses were stratified by gender and site of origin.

**Results:**

A significant linear association was found between the mean scores of the Short Physical Performance Battery and ordinal categories of self-rated health across research sites and gender groups. After extensive control for objective physical and mental health indicators and socio-demographic variables, these graded associations became non-significant in some research sites.

**Conclusion:**

These findings further confirm the validity of SRH as a measure of overall health status in older adults.

## Introduction

Physicians usually initiate consultations with open-ended questions such as, “How are you?” or, “How are you feeling?” These questions address patients’ perception of their overall health status, a concept also known as self-rated health (SRH). However, the validity of SRH has been a matter of debate in epidemiological and clinical research [1], due to its uncertain biological significance [2]. Some aspects of SRH remain open to debate, notably the biological significance of rating oneself with poor or very poor health. A number of questions arise nowadays regarding this issue, such as: In terms of biological correlates, what is the significance of answers to global questions about health status, such as ‘How would you rate your health’? How could these global health assessments be used in daily clinical work? How are these subjective judgments influenced by personal bias as opposed to objective health criteria?

SRH has become a well-established predictor of mortality and other adverse outcomes [3–9]. The predictive value of SRH appears to be independent of other well-known factors associated with mortality, such as cardiovascular disease and smoking, among others. In spite of its widespread use in different health surveys, there is a lack of information concerning the consistency of perceptions about SRH across populations in different cultural and socioeconomic contexts [10–13].

Physical performance tests also have good predictive value in terms of mortality, disability, and rate of hospitalization, and appear to be consistent across populations, due to their objectivity and the possibility of reliable third-party measurement [14]. These tests include a variety of measures, including grip strength, gait speed, endurance, balance, and others. In particular, the Short Physical Performance Battery (SPPB) consists in a set of three tests that assess gait speed, balance, and strength. It accurately predicts adverse outcomes such as mobility disability [15, 16], ADL disability, hospitalization, nursing home admission, frailty, and mortality [17–20]. In addition, the validity of the SPPB has been confirmed in studies conducted in Brazil and Colombia [21–23]. Among the SPPB tests, gait speed is the most widely used and studied for older adults, and has a clear association with mortality and other adverse outcomes [24, 25]. Moreover, SRH and gait speed seem to have an additive prognostic significance, as shown by Cesari et al. who found that Mexican-American older adults with both low SRH and low gait speed had the strongest association with mortality [26].

Since both SRH and SPPB have a graded prediction of mortality (the higher the score, the lower the probability of dying), we would expect physical performance to be associated with SRH. In fact, slow gait speed and difficulty in walking were associated with poor SRH in cross-sectional analyses of the Women's Health and Aging Study, conducted on a sample of mobility-disabled women in the United States [27]. However, to our knowledge no study has shown the association between SRH and the SPPB in diverse populations of older men and women. Such a study could demonstrate the validity of SRH as a measure of global health status that aligns with objective indicators of physical function among older adults in different cultural settings. In addition, it could provide evidence of the significance of SRH in clinical settings.

Therefore, the aim of this study is to explore the validity of SRH across different populations of older men and women, by comparing it to the SPPB.

## Materials and Methods

### Settings and Design

The International Mobility in Aging Study (IMIAS) is a multi-national cohort study, whose main purpose is to examine the dynamics of mobility disability among older adults across different settings. Current participating sites are: Natal (Brazil), Manizales (Colombia), Tirana (Albania), Saint-Hyacinthe (Quebec, Canada), and Kingston (Ontario, Canada). The full description of this study is available elsewhere [19]. Briefly, older adults between 65 and 74 years old and without severe cognitive decline were included.

Severe cognitive decline was defined as four or more errors in the orientation scale of the Leganes Cognitive Test (LCT) [28]–fewer than 5 people were excluded for this reason at each site. In Tirana, Manizales, and Natal, recruitment began in 2012 using random selection from the lists of patients registered at neighbourhood health centres (200 women and 200 men between 65 to 74 years of age). In Natal and Tirana, more than 90% of people in that age group are registered in the national healthcare systems, which provide universal coverage. In Manizales, around 70% of people in that age group are registered in the public healthcare system. In the Canadian sites, the local ethics committees did not allow the research team to contact potential participants directly. Instead, in Kingston and Saint-Hyacinthe a letter from primary care physicians invited potential participants wishing to participate in the study to contact our field coordinator. Response rates were higher than 90% in Manizales and Natal. At the Canadian sites, 95% of the subjects who called our field coordinator participated in the study. Once recruitment was complete, a trained team carried out face-to-face interviews with participants using the same tools that had been previously adapted and validated at each site.

### Measurements

#### Outcome

The SPPB is composed of three timed components to assess balance, gait speed, and lower limb force [29]. Each component is scored from 0 to 4 points, with subjects not able to perform a test scored at zero, and four representing the best category of performance. The final score is the sum of the three tests, ranging from a minimum of zero points to a maximum of 12; a cut-off point of less than 8 points was used to identify those with poor physical function [21, 22].

#### Predictor

SRH was collected using a five-category single question: “How would you say is your health: very good, good, fair, poor, or very poor?” For the bivariate analyses, we combined the ‘poor’ and ‘very poor’ categories due to low frequency of very poor health, particularly in the two Canadian cities. For the multivariate analyses we further collapsed the ‘very good’ and ‘good’ categories due to the low frequency of the ‘very good’ category in Tirana and Natal. Thus, SRH was recoded into three categories for multivariate analyses: ‘good/very good’, ‘fair’, and ‘poor/very poor’ (see below), with ‘fair’ as the reference category.

#### Confounders

According to the relevant literature the following variables were identified as potential confounders: age, sex, marital status, years in school (<7 years, 7–11 years, and ≥12 years in school), number of chronic conditions, depressive symptoms and cognitive status, height and weight. These variables, which have previously been associated with SRH [30, 31] and physical performance, are not likely to be in a causal pathway between physical performance and SRH [32, 33]. Marital status was categorized as single (never married), married, widower, or divorced/separated. Older adults were asked if they had ever been diagnosed with any of these seven chronic conditions: diabetes, cancer, lung disease, heart disease, stroke, arthropathy, or osteoporosis. A comorbidity index was created measuring the number of these diseases reported, with a range from 0 to 7.

Cognitive function, depressive symptoms, and body composition were analyzed as continuous variables. Cognitive function was tested using the LCT, a screening test for dementia with known validity in populations with low education [28]. Depressive symptoms were reported as the score on the Center for Epidemiologic Studies Depression Scale, a widely used 20-item test that recognizes a wide array of depressive symptoms in older adults. The lowest score possible is zero and the highest 60 (highest possible burden of depressive symptomatology) [34]. Finally, body mass index (BMI)–estimated as weight in kilograms divided by squared height in meters–was also included as a confounding variable.

### Statistical analyses

Descriptive statistics stratified by sex and sites were reported. To test significance of the differences between sites (for outcome, predictor, and confounding variables), one-way analyses of variance (ANOVA) or Chi Square were used, as appropriate. ANOVA models were used to test linear trends of SPPB according to three SRH categories by groups of sex and site. The F-statistic was used to test these trends and generate p-values. Sex- and site-specific logistic regressions were fitted to examine the associations between low physical performance (SPPB less than 8) and SRH (using ‘fair’ as reference category). Two models were used, the first adjusted only for age and the second adjusted for age, years in school, marital status, LCT, CES-D, BMI, and number of comorbidities. Odds ratios were estimated with 95% confidence intervals (CI) for all models. The data were analyzed using STATA version 13.1.

### Ethical considerations

The IMIAS project received ethics approval from the Research Centre of the University of Montreal (CR-CHUM), the Albanian Institute of Public Health, and the research ethics boards of the Federal University of Rio Grande do Norte (Brazil), Queen’s University (Kingston), and the University of Caldas (Columbia). All study participants provided written informed consent and were told they could withdraw at any time. In Brazil and Colombia, the consent form was read to participants who could not read and write, after which oral consent was obtained and the participants placed a mark on the form to acknowledge agreement. Oral consent was only used for those who could not read or write; extensive explanation of the protocol was provided by field researchers; and two witnesses (related to neither the participants nor the researchers) who were present for the procedure signed a document testifying that thorough informed consent had been given by the participants, that they had understood, and that all questions raised were fully answered. The participants then made a mark (usually a cross) in the signature space or, if that was not possible, gave their thumbprint. As stated previously, the procedure was approved by the appropriate ethics committees of participant sites.

## Results

Of a total of 1,995 older adults assessed, 52.1% (n = 1,040) were women (without significant differences between sites). Mean age was 69.1 (±2.6 standard deviations [SD]) overall and was significantly higher in non-Canadian sites (p < .001) (see Table 1). Regarding years in school, 65.7% of participants had 7 years or more; in non-Canadian sites, women were less educated than men. The majority of participants were married (67.5%), and the mean number of chronic conditions was not significantly different across sites (p = .73) (overall mean 1.8 [±1.32 SD]). LCT scores were not different between sites, 27.9 (±3.8 SD); and the overall mean for CES D was 11.02 points (±11.02 SD). The overall mean height of men was 1.67m (±.08 SD), with no differences between sites; the mean height of women was 1.54m (±.07 SD), with significant difference between sites (p < .001), the lowest height being observed in women of Natal and Manizales (1.5m).

**Table 1:** General Characteristics of the Sample According to Site and Stratified by Sex.

	Kingston		Saint-Hyacinthe		Tirana		Manizales		Natal	
	Men (n = 186)	Women (n = 212)	Men (n = 191)	Women (n = 210)	Men (n = 188)	Women (n = 206)	Men (n = 198)	Women (n = 202)	Men (n = 192)	Women (n = 210)
Age, mean (SD)	69 (2.7)	69.1 (2.6)	68.6 (2.7)	68.5 (2.6)	69.6 (3.2)	69.1 (3)	69.2 (2.9)	69.3 (3)	69.1 (2.7)	69.2 (2.6)
Years in school, n(%)										
<7 years	2 (1)	0 (0)	14 (7.3)	14 (6.7)	15 (7.9)	30 (14.5)	138 (70)	157 (77)	135 (70)	178 (84)
7–11 years	21 (11.2)	23 (10)	59 (30.8)	82 (39)	83 (44.1)	105 (50)	30 (15.1)	37 (18.3)	42 (21.8)	25 (11.9)
≥12 years	163 (87)	189 (89)	118 (61)	114 (54)	90 (47.8)	71 (34.4)	30 (15.1)	8 (3.9)	15 (7.8)	7 (3.3)
Marital status, n (%)										
Single	6 (3.2)	9 (4.2)	6 (3.1)	16 (7.6)	1 (.5)	3 (1.4)	20 (10.1)	33 (16)	9 (4.69)	19 (9)
Married	155 (83)	116 (55)	160 (84)	132 (62)	177 (94)	129 (62)	140 (70)	67 (33.1)	167 (87)	105 (50)
Widow	8 (4.3)	44 (20)	8 (4.1)	25 (11.9)	9 (4.7)	70 (33)	14 (7)	81 (40)	6 (3.1)	64 (30.4)
Divorced	17 (9.1)	43 (20)	17 (8.9)	37 (17.6)	1 (.5)	4 (1.9)	24 (12.1)	21 (10.4)	10 (5.2)	22 (10.5)
Comorbidity, mean (SD)	1.74 (1.2)	1.9 (1.3)	1.54 (1.2)	1.91 (1.4)	1.96 (1.2)	2.58 (1.3)	1.18 (1.1)	1.81 (1.3)	1.52 (1.1)	2.41 (1.3)
LCT score, mean (SD)	30.4 (2.1)	30.5 (2.3)	28.8 (1.9)	29.1 (2.4)	27.7 (3.3)	26.7 (4.4)	25 (5)	25.5 (4.9)	27.7 (2.6)	27.6 (2.7)
CESD score, mean (SD)	5.3 (6.1)	7.6 (8.6)	11.3 (9.8)	12.4 (10.2)	12.5 (9.6)	17.7(13.1)	9.5 (7.9)	12 (9.4)	7.3 (5.9)	13.5 (9.8)
Height in meters, mean (SD)	1.7 (.1)	1.6 (.1)	1.7 (.1)	1.5 (.1)	1.6 (.1)	1.5 (.1)	1.6 (.1)	1.5 (.1)	1.6 (.1)	1.5 (.1)
Weight in kilograms, mean (SD)	84.9(14.7)	72.6(16.4)	82.6(13.9)	68.4 (14.2)	79.3 (11.6)	70.5(12.7)	67.5 (11.2)	60.4(10.2)	71.7 (12)	65.1 (13.7)
BMI in kilograms per square meter, mean (SD)	27.9 (4.7)	28 (6.1)	28.5 (4.8)	27.6 (5.6)	28.2 (3.9)	29.7 (4.9)	25.2 (3.6)	26.8 (4.2)	26.6 (3.8)	28.6 (5.9)
SPPB, mean (SD)	10.3 (1.6)	10.1 (2)	10.4 (1.5)	9.9 (1.7)	9.7 (2.3)	8.3 (2.9)	9.1 (3.1)	9 (2.7)	9.7 (2.2)	8.4 (2.3)
SPPB <8, n (%)	13 (6.9)	18 (8.4)	9 (4.7)	18 (8.5)	23 (12.2)	59 (28.6)	29 (14.6)	37 (18.3)	32 (16.6)	58 (27.6)
SRH, n (%)										
Very good	74 (39.7)	104 (49)	70 (36.6)	60 (28.5)	9 (4.7)	4 (1.9)	24 (12.1)	27 (13.3)	8 (4.1)	3 (1.4)
Good	89 (47.8)	74 (34.9)	90 (47.1)	111 (52)	68 (36.1)	56 (27.1)	85 (42.9)	65 (32.1)	60 (31.2)	46 (21.9)
Fair	19 (10.2)	27 (12.7)	29 (15.1)	34 (52.8)	89 (47.3)	113 (54)	76 (38.3)	97 (48)	103 (53)	118 (56)
Poor/Very poor	4 (2.1)	7 (3.3)	2 (1.1)	5 (2.3)	22 (11.7)	33 (16.1)	13 (6.5)	13 (6.4)	21 (10.9)	43 (20.4)

n = number; SD = Standard Deviation; LCT = Leganés Cognitive Test; CES D = Center of Epidemiology Studies Depression Scale

BMI = Body Mass Index; SPPB = Short Physical Performance Battery

There was a significant difference in SPPB score across sites: the overall mean was 9.5 (±2.4 SD), with the lowest score in Manizales (9.04, ±2.9 SD), and the highest in Kingston (10.2 ±1.8 SD) (p < .0001). With the exception of Kingston and Manizales, there were also significant differences in SPPB total scores between men and women at each site.

Poor physical performance was defined as a SPPB score below eight. Poor physical performance was observed in 14.8% (n = 296) of the full sample, with the highest proportion of older adults below this threshold in Tirana (30.4%, n = 90) and the lowest in Saint-Hyacinthe (9.1%, n = 27) (p < .0001).

SRH was distributed as follows: 383 (19.2%) participants reported their health as ‘very good’, 744 (37.2%) as ‘good’, 705 (35.3%) as ‘fair’, and 163 (8.1%) as ‘poor/very poor’, with significant differences between all the sites. In Manizales, Natal, and Tirana, poor/very poor perceptions of health were reported by 20% of the population. The site with the highest proportion of very good/good health was Kingston, and the site with the highest proportion of poor/very poor health was Manizales. In addition, women reported poor and very poor health more frequently than men, but only in the non-Canadian sites (p = .33). At the Canadian sites, there was no significant difference in self-rated health between men and women (p = .24).

A significant inverse gradient in physical performance was observed with respect to categories of self-rated health in all analyses by sex and city (p < .0001) (Table 2). Among men, a steep gradient was observed at each site, going from a mean SPPB score of 11.1 (±.9 SD) for SRH ‘very good’ in Tirana to 10.3 (±1.9) in Natal. The mean SPPB scores of women with ‘very good’ SRH ranged from 11.2 (±.9 SD) in Tirana to 9.2 (±3.1 SD) in Manizales. The lowest mean SPPB score for men in the SRH category of ‘poor/very poor’ was 5.5 (±.7 SD) in Saint-Hyacinthe, while the highest score in the same category was 8 (±2.7 SD) in Natal. For women, the lowest mean SPPB score for the SRH category of ‘poor/very poor’ was 5.7 (±3.1 SD) in Kingston, and the highest was 7.6 (±2.3 SD) in Natal.

**Table 2:** Mean (SD) of SPPB^†^ According to Self-Rated Health (Four Categories) at Each Site and Stratified by Sex^†^.

Site	Kingston				Saint-Hyacinthe				Tirana				Manizales				Natal			
Sex	Men		Women		Men		Women		Men		Women		Men		Women		Men		Women	
SRH	N	Mean (SD)	N	Mean (SD)	N	Mean (SD)	N	Mean (SD)	N	Mean (SD)	N	Mean (SD)	N	Mean (SD)	N	Mean (SD)	N	Mean (SD)	N	Mean (SD)
Very good	74	10.7 (1.5)	104	10.8 (1.1)	70	10.9 (1.1)	60	10.4 (1.2)	9	11.1 (.9)	4	11.2 (.9)	24	10.5 (1.1)	27	9.2 (3.1)	8	10.3 (1.9)	3	9.3 (1.1)
Good	89	10.2 (1.6)	74	9.7 (2.2)	90	10.5 (1.4)	111	10.1 (1.4)	68	10.6 (1.3)	56	9.5 (2.2)	85	9.1 (3.5)	65	9.4 (2.9)	60	10.1 (2.1)	46	8.9 (2.3)
Fair	19	9.7 (1.5)	27	10.1 (1.8)	29	9.2 (1.5)	34	8.9 (2.2)	89	9.6 (2.5)	113	8.3 (2.8)	76	9 (2.7)	97	8.8 (2.4)	103	9.9 (2.1)	118	8.5 (2.3)
Poor/Very poor	4	7.7 (.9)	7	5.7 (3.1)	2	5.5 (.7)	5	6.4 (2.6)	22	7.4 (2.7)	33	6 (3.1)	13	7 (4)	13	7.5 (2.1)	21	8 (2.7)	43	7.6 (2.3)

SRH = Self-rated Health; N = Number; SD = Standard Deviation; SPPB = Short Physical Performance Battery

^†^ F-test for linear trend used to generate p-value; all p-values < .001

The results of the multiple logistic regression models are shown in Table 3. Among men, reporting poor/very poor health was significantly associated with poor SPPB at all sites except for Manizales, after controlling for age (Model 1). After extensive adjustment for socio-demographic variables, chronic conditions, body mass index, cognitive function, and depressive symptoms, these associations were attenuated and lost significance at all sites except at Saint-Hyacinthe (Model 2).

**Table 3:** Multiple Logistic Regression Models for SPPB as Dependent Variable (<8 points) and Self-rated Health in Three Categories (Good/Very Good, Fair [reference], and Poor/Very Poor) as Independent Variable Stratified by Sex.

		Model 1 OR (95% CI)				Model 2 OR (95% CI)			
Site	SRH	Men	p-value	Women	p-value	Men	p-value	Women	p-value
Kingston	Very good/Good	.6 (.1–3.2)	.576	.4 (.1–1.8)	.298	1.1 (.1–9)	.897	.5 (.1–2.5)	.482
	Fair	Reference		Reference		Reference		Reference	
	Poor/Very Poor	25.1 (1.5–396)	.022	10 (1.5–73)	.016	14.3 (.54.07)	.118	4 (.4–40)	.238
Saint- Hyacinthe	Very good/Good	.06 (.1-.3)	.001	.1 (.03-.3)	< .001	.06 (.01-.6)	.018	.2 (.04-.9)	.038
	Fair	Reference		Reference		Reference		Reference	
	Poor/Very Poor	6.3 (4.3–10.1)	.001	3.9 (.5–28.5)	.175	6.2 (4–9.8)	.001	1 (.04–9)	.967
Tirana	Very good/Good	.07 (.01-.6)	.015	.2 (.1-.6)	.007	.1 (.01–1.4)	.103	.4 (.1–1.1)	.09
	Fair	Reference		Reference		Reference		Reference	
	Poor/Very Poor	4.04 (1.4–11.4)	.008	3.6 (1.6–8.1)	.002	4.2 (.9–20.1)	.068	3.3 (1.3–8)	.019
Manizales	Very good/Good	.7 (.3–1.7)	.468	.5 (.2–1.3)	.194	.9 (.3–2.7)	.967	1.1 (.4–2)	.844
	Fair	Reference		Reference		Reference		Reference	
	Poor/Very Poor	2.5 (.6–9.6)	.179	2.6 (.7–9.4)	.124	3.5 (.6–18.7)	.143	2 (.5–8.2)	.276
Natal	Very good/Good	.9 (.3–2.2)	.855	1.06 (.2–1.4)	.267	1.4 (.5–4)	.445	1 (.3–2.6)	.999
	Fair	Reference		Reference		Reference		Reference	
	Poor/Very Poor	4.7 (1.5–14.05)	.006	3.1 (1.4–6.6)	.003	2.9 (.8–10.2)	.091	2.6 (1.1–6)	.026

OR = Odds Ratio; CI = Confidence Interval; SRH = Self-rated Health SPPB = Short Physical Performance Battery

**Model 1**. Adjusted for age.

**Model 2.** Adjusted for age, years in school, marital status, Leganes Cognitive Test score, CES D, body mass index, and comorbidity

Among women, large significant associations were observed in Kingston, Tirana, and Natal between poor/very poor SRH and poor physical performance; these associations were also strong in Saint-Hyacinthe and Manizales, but did not reach statistical significance. After adjustment for all covariates, associations became non-significant for women living in Manizales and the Canadian cities, but they remained strong and significant for women in Tirana and Natal.

## Discussion

These findings further confirm the validity of SRH as a measure of overall health status in older adults. According to our results, the prevalence of poor physical function increased as perceptions of health worsened, for both men and women and across all five research sites. Associations remained when controlling for age, but became attenuated or disappeared after extensive adjustments for education and objective indicators of physical and mental health. Even across different cultural backgrounds, when compared with a robust reference standard such as the SPPB, SRH proved to be an effective and valid global measurement tool for poor health and physical function.

The fact that associations tended to disappear when adjusting for socioeconomic and objective physical and mental health indicators further strengthens the validity of the concept, since these adjustments show that SRH is capturing important aspects of health and socioeconomic status that explain the pathway from SRH to physical performance. Although adverse conditions can exacerbate the perception of a poor SRH, the association persists when these conditions are not present. However, women living in Natal and Tirana who stated their health was poor or very poor continued to be more likely to have poor physical function, indicating that additional aspects influencing SRH have not been taken into account among women of these two cities. Importantly, it is these same women who had the worst perceptions of health and the poorest function among all sex and city comparison groups. However, adding more variables to the models might have resulted in loss of power.

Thus, when a clinician asks a patient to rate his/her health and the patient indicates it is ‘poor’ or ‘very poor’, the clinician can be fairly certain that the likelihood of poor physical function is high; that potential adverse outcomes such as mobility disability, hospitalization, and death are likely outcomes for that particular older adult; and that the meaning of this self-reported status is fairly consistent across countries. This is very clear, as it was true for all groups of men and women at each of the five research sites. Admittedly, some of the differences when rating own health could be solely attributed to country of origin (sociocultural background) and education, or to rating different constructs of health (e.g., a specific disease versus overall physical health) [35]. Nevertheless, low SRH should prompt the clinician to search for chronic conditions that could be affecting the patient’s perception.

Another contribution of this study is its focus on the age span of 65 through 74. This is a very dynamic period in disability transitions and a key age range for prevention of mobility loss and disability. Many people in this age group are also going through retirement from the work force. It is a very opportune time for health behaviour changes that can delay further physical decline. However, this strength of the study could also represent a limitation, since some literature suggests that the predictive value of SRH for mortality diminishes as people become older, and therefore the same might be true of the predictive value of SRH for physical function [36].

In clinical settings, the ease of obtaining SRH may diminish the need to run unnecessary tests that could be harmful to older adults, and in particular to those in the frail spectrum of health. We provide evidence that a single question on health status perception could have enormous implications for the care of older adults. Even where SRH cannot replace other diagnostic tools, it could help in decision making, especially in lower and middle-income countries, where resources are scarce [37].

The weakest associations of poor SRH with poor function, found among men and women in Manizales, could be due to cultural lack of acceptance or to lower recognition of being in poor health. We have already reported that older adults in Manizales tended to report better health than older adults of Natal within each level of mobility, as assessed by the videos of the Mobility Assessment Tool-Short Form [33]. However, even with a lack of recognition of poor health in Manizales, the reports of poor health had validity in the unadjusted analyses (Table 2). Social inequality has been shown to impact physical performance, particularly in early old age, an aspect that also needs to be taken into account when interpreting our results [27].

This study has limitations: the sample size for each strata by sex and city is one of the most important–as shown by the large confidence intervals of the associations, particularly in Model 2. We found no substantial differences when we carried out sensitivity analyses using SPPB cut-off points of less than 9 and less than 10, as appropriate–as was done in the recently published Lifestyle Interventions and Independence for Elders LIFE [38] clinical trial. However, this also points to the need for cut-off points tailored to each population, which has been demonstrated in the case of other physical performance tests [39]. Notwithstanding, with a larger sample size we would have been able to estimate the associations of poor physical function across the five levels of SRH precisely, to construct ROC curves at each site, and to validate the cut-off points for poor physical function for each sex and city group, resulting in a fuller investigation of the association under study. In addition, SRH and SPPB should be compared with other tools that assess physical function, are widely accepted, and also have standardized methodology in their application and interpretation (e.g., the World Health Organization’s International Classification of Function). Finally, while one might think that having to collapse the categories of SRH due to limited sample size might have reduced the accuracy of our construct, in actuality patients in clinical practice used few categories when answering this type of question. Therefore, having three categories in our analyses might in fact better represent the real clinical situation and the actual answers of older adults. Further research will help confirm and expand on the results obtained in this manuscript.
